# Simultaneous Determination of the Main Phenolic Compounds and Triterpenic Acids in Table Olives Using RP-UHPLC-DAD Chromatography

**DOI:** 10.3390/molecules31173029

**Published:** 2026-08-28

**Authors:** Aggeliki Kalogeropoulou, Vasileios Zavrakoglou, Fani Th. Mantzouridou, Nikolaos Nenadis

**Affiliations:** Laboratory of Food Chemistry & Technology, School of Chemistry, Aristotle University of Thessaloniki, 541 24 Thessaloniki, Greecefmantz@chem.auth.gr (F.T.M.)

**Keywords:** table olives, UHPLC-DAD, phenolic compounds, hydroxytyrosol, oleuropein, triterpenic acids, analytical methodology

## Abstract

A rapid reversed-phase ultra-high-performance liquid chromatography method with diode-array detection (RP-UHPLC-DAD) was developed and validated for the simultaneous determination of major bioactive phenolic compounds (hydroxytyrosol, tyrosol, and oleuropein) and triterpenic acids (maslinic and oleanolic acids) in table olives from major Greek cultivars. Existing chromatographic methods for table olives typically target either the phenolic compounds or the triterpenic acids, requiring separate protocols. The proposed method determines both classes simultaneously, resolving all five analytes (resolution, R_s_ ≥ 5) within a 20 min separation (25 min total run) using a single methanolic co-extraction, thereby reducing analysis time and increasing throughput relative to sequential approaches. This protocol was selected among four evaluated extraction procedures, based on simplicity and efficient analyte extraction. The method showed excellent linearity (R^2^ > 0.999), along with satisfactory sensitivity, selectivity, accuracy, and precision across the tested ranges. Recoveries of 70.8–108.9% were obtained in three different matrices (Chalkidiki, Kalamata, and Throumba Thassou olives), indicating satisfactory applicability to complex foods. The samples differed in their analyte profiles, with hydroxytyrosol predominating in the Chalkidiki and Kalamata samples, whereas oleuropein was detected only in Throumba Thassou olives. Sustainability assessment using the Complementary Green Analytical Procedure Index (ComplexGAPI) indicated moderate greenness, while a Blue Applicability Grade Index score of 72.5 denoted good practical applicability.

## 1. Introduction

Table olives have been a major plant-derived fermented food in the Mediterranean area for centuries and have progressively evolved into a high-value commercial product consumed worldwide [1]. World table olive production and consumption reached approximately 2.8 and 2.86 million tons, respectively, in the 2023/24 crop year [2], underlining their widespread dietary presence. Although their relatively high sodium content, resulting from brining processing, warrants dietary consideration, table olives can serve as a vehicle for delivering to consumers various bioactive compounds [3]. These include phenolic compounds (hydroxytyrosol, tyrosol, and oleuropein) which exhibit antioxidant and cardioprotective properties [4], and pentacyclic triterpenic acids (maslinic and oleanolic) which demonstrate antimicrobial, anti-inflammatory, and anticancer activities [5,6].

Raw olives require processing such as Spanish-style (alkaline treatment/brine fermentation), Greek-style (direct brining/fermentation), or Californian-style (air oxidation) methods or natural maturation on the tree (e.g., Throubolia cultivar in Greece) to reduce bitterness primarily caused by oleuropein and to render the fruit edible. These processing approaches significantly influence the levels of bioactive compounds, particularly phenolic constituents, through enzymatic, chemical, and diffusional mechanisms. Alkaline (Spanish-style) treatment hydrolyses oleuropein to hydroxytyrosol, whereas natural fermentation and on-tree maturation proceed more slowly, with phenolic losses occurring mainly by diffusion into the brine, largely retaining the original profile. In contrast, pentacyclic triterpenic acids are mainly influenced by alkaline treatments, which reduce their content through solubilisation into the alkaline solutions, rather than fermentation-driven transformations [7,8,9,10,11,12].

The diversity of olive cultivars, processing technologies, agronomic practices, and geographical origins results in a wide range of commercial table olive products exhibiting highly variable bioactive compound profiles. This variability highlights the need for validated analytical protocols to support quality control and substantiation of health-related claims. Several validated high-performance liquid chromatography (HPLC) methods using diode-array (DAD) or mass spectrometric (MS) detection have been reported for the determination of phenolic compounds in table olives from some Greek [13,14,15], Spanish [13,14,16,17], and American cultivars [14]. Pentacyclic triterpenic acids have also been investigated in table olives using chromatographic techniques; however, only one fully validated analytical method has been reported to date, based on MS detection and applied to Marfil natural table olives [18].

Simultaneous determination of phenolic compounds and pentacyclic triterpenic acids remains analytically challenging due to their markedly different polarities (Table S1), which often necessitates separate extraction and chromatographic protocols [11,12]. Recent studies have explored analytical approaches for the simultaneous determination of both compound classes in olive-derived matrices. Moreno-González et al. [19] validated the extraction of three pentacyclic triterpenes and sixteen polyphenols in Arbequina and Empeltre table olives; however, analysis required separate chromatographic runs using LC with atmospheric pressure chemical ionization (APCI)-MS for triterpenes and LC with electrospray ionization (ESI)-MS/MS for polyphenols due to distinct ionization requirements. Olmo-García et al. [20] reported comprehensive metabolite profiling of olive oil and olive flour, identifying fifty-seven metabolites, including phenolics, triterpenes, and tocopherols, using MS detection (ESI+ mode) with sub-2 μm particle columns and a total analysis time of 31 min. Similarly, Xie et al. [21] developed a simultaneous extraction and LC-DAD-MS (ESI) method for various olive tissues and olive oil by-products, although the total chromatographic run time was 70 min. While MS-based methods provide excellent sensitivity and structural confirmation, their practical application in routine quality control may be limited by high instrumentation costs, complex data processing requirements, and, in some cases, extended analysis times that restrict analytical throughput. Conversely, DAD offers lower selectivity than MS, particularly at the low wavelengths (~210 nm) required for compounds lacking extended chromophores such as triterpenic acids, where co-eluting matrix constituents may also absorb. Reliable quantification by DAD, therefore, depends on adequate chromatographic separation combined with complementary spectral criteria.

In this context, the present study aimed to develop and validate a rapid reversed-phase ultra-high-performance liquid chromatography method with diode-array detection (RP-UHPLC-DAD) targeting five major bioactive markers (hydroxytyrosol, tyrosol, oleuropein, maslinic acid, and oleanolic acid) for routine quality control of table olives. The method employs a single methanol-based extraction protocol, selected after comparative screening of four established extraction procedures, and uses a 2.6 μm particle column compatible with both UHPLC and conventional HPLC systems. Chromatographic separation was achieved within 20 min (25 min total run including re-equilibration) in a single chromatographic run. Comprehensive method validation was performed, with particular emphasis on addressing analytical challenges associated with UV detection of triterpenic acids at 210 nm, a wavelength commonly applied in table olive triterpene analysis [7,11] and in validated chromatographic methods for triterpenic compounds in botanicals of pharmaceutical relevance [22,23].

Three major Greek table olive cultivars—Chalkidiki green olives, Kalamata black olives, and Throumba Thassou black olives processed using Spanish-style fermentation, Greek-style fermentation, and natural on-tree maturation, respectively—were included in both method validation and application. These distinct processing approaches generate chemically diverse olive matrices, enabling evaluation of method performance across a range of table olive processing conditions and compositional profiles. Since cultivar and processing style are inherently linked in these commercial products, their effects cannot be separated. The environmental performance of the developed method was assessed using the Complementary Green Analytical Procedure Index (ComplexGAPI) [24], while practical applicability was evaluated using the Blue Applicability Grade Index (BAGI) [25], providing a comprehensive assessment of sustainability and operational suitability for routine industrial implementation. Overall, the proposed method complements MS-based analytical approaches by providing an accessible and cost-effective tool for targeted monitoring of established bioactive markers in laboratories lacking access to (U)HPLC–MS instrumentation.

## 2. Results and Discussion

### 2.1. Elution Protocol

The three selected phenolic analytes (hydroxytyrosol, tyrosol, and oleuropein) are considerably more polar than the two pentacyclic triterpenic acids, as reflected in their calculated partition coefficient (log *P*) values (see Supplementary Table S1). This marked polarity difference explains why methods targeting triterpenic acids alone typically employ isocratic elution with a high proportion of organic solvent (e.g., 92% methanol) [12], whereas the simultaneous determination of phenolic compounds and triterpenic acids generally requires substantially longer runs [21].

To reduce analysis time while maintaining adequate chromatographic performance, an Accucore C18 column (100 mm × 4.6 mm, 2.6 μm solid-core particles, Thermo Fisher Scientific, Waltham, MA, USA) was selected. This column, which is compatible with both UHPLC and HPLC platforms, is designed for optimal retention through hydrophobic interactions across a broad range of nonpolar analytes [26]. Acetonitrile was selected as the organic modifier instead of methanol due to its higher elution strength, which enabled a substantial reduction in run time from 70 min [21] to 25 min in the present study. Although acetonitrile has a higher National Fire Protection Association (NFPA) health hazard rating (3) compared to methanol (2), it offers lower baseline noise and a shorter UV cutoff (190 vs. 205 nm), both of which are advantageous for analytes exhibiting low absorption maxima such as triterpenic acids monitored at 210 nm [27].

**Figure 1:**
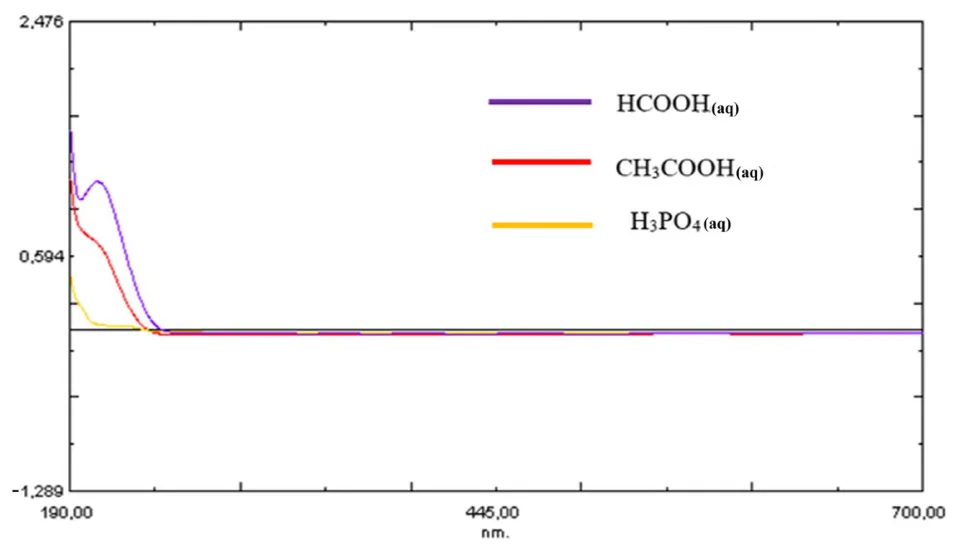
UV spectra of aqueous solutions of formic, acetic, and phosphoric acids at 210 nm.

Initially, the elution program reported by Xie et al. [21], which employed acetonitrile as the organic phase, was adapted to the selected column. However, when applied to a standard mixture of the five target analytes, the gradient resulted in abrupt baseline drops during the stepwise increase of acetonitrile content, thereby compromising the detection and quantification of triterpenic acids (see Supplementary Figure S1). This behaviour originated from the formic acid in the aqueous mobile phase, which at the recommended pH of 3.2 caused significant absorption at 210 nm (Figure 1).

Because baseline absorbance was zeroed prior to the gradient elution, the progressive introduction of acid-free acetonitrile in the mobile phase during the run led to the appearance of negative absorbance signals. A similar effect was observed when acetic acid was used. Substitution of formic or acetic acid with phosphoric acid (0.2% *v*/*v*, resulting in pH ~2) eliminated this problem, as the aqueous mobile phase containing the latter exhibited negligible absorbance at 210 nm, in accordance with Snyder et al. [27], who recommend acetonitrile-phosphate mobile phases for routine detection at 200 nm (10 mM phosphoric acid has a UV cut-off <200 nm). Although a strong acid, phosphoric acid is non-flammable and chemically stable, with an NFPA profile comparable to formic and acetic acids. Its non-volatility does not constitute a limitation, as DAD rather than mass spectrometric detection was used.

**Figure 2:**
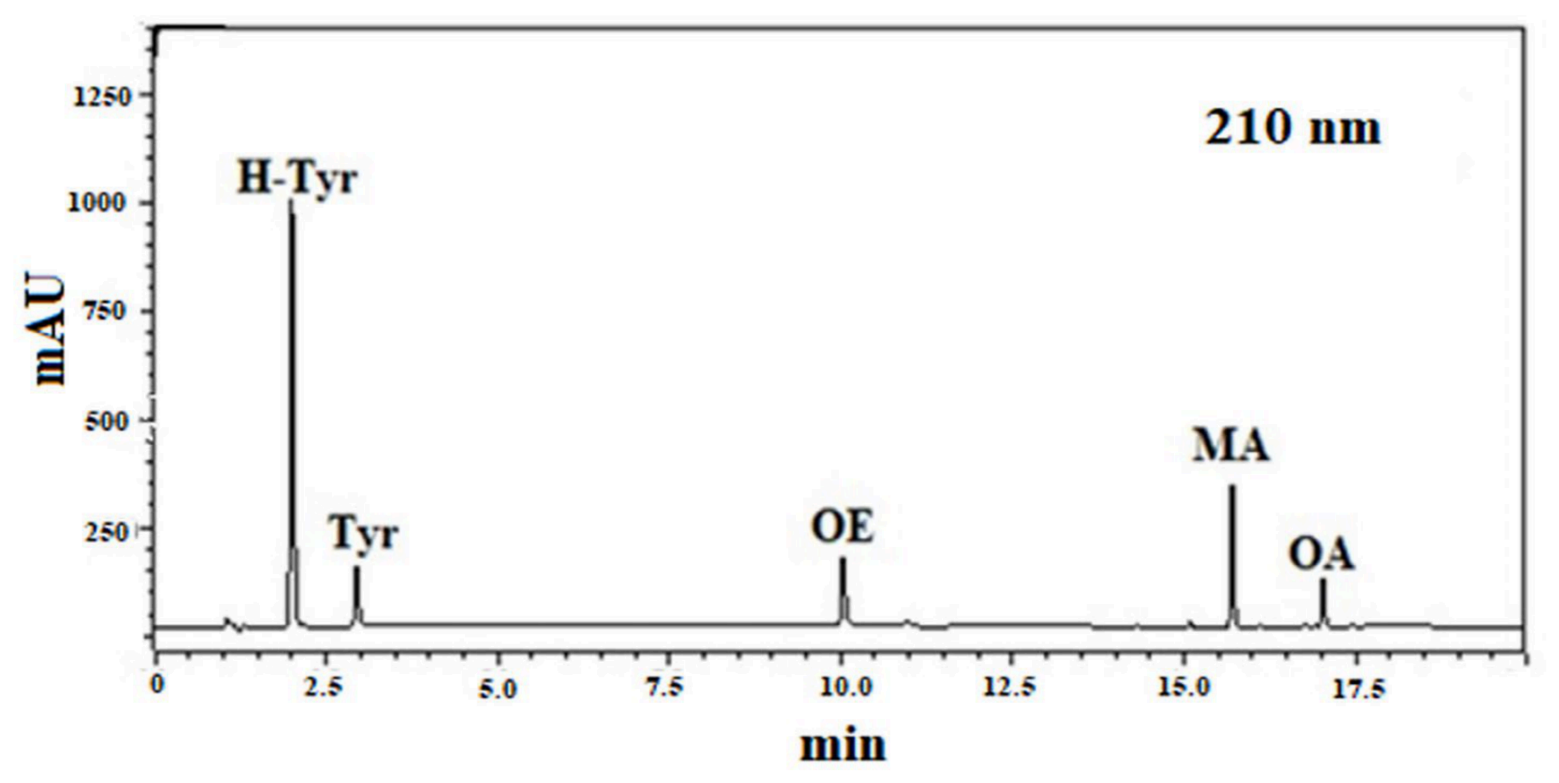
Chromatogram of the five target analytes (reference compound mix) recorded at 210 nm under the optimized conditions.

After optimization, the final gradient protocol achieved complete elution of all five target analytes within 20 min (25 min total run including re-equilibration) (Figure 2). Compared with the method reported by Xie et al. [21], the optimized protocol reduced run time by 3.5-fold and solvent consumption by 1.75-fold.

System suitability was verified using standard mixtures of the five analytes. Retention times were reproducible across concentration levels. All target analytes were baseline-resolved, with resolutions between adjacent analyte pairs ranging from 5.4 to 39.1 (United States Pharmacopeia, USP convention), and peak symmetry was acceptable (Table 1). Theoretical plate counts are not reported, as the method employs gradient elution, under which N does not provide a meaningful measure of column efficiency.

### 2.2. Comparison of Extraction Protocols

Four extraction protocols previously reported in the literature and coupled to RP-HPLC-DAD analysis of phenolic compounds and/or triterpenic acids were comparatively evaluated using a raw (unprocessed) Chalkidiki olive sample, freeze-dried and pulverized prior to extraction, to identify the most suitable one for the co-extraction of the target analytes. The evaluated protocols [12,17,21,28] differed in solvent composition, extraction time, and sample-to-solvent ratio, as summarized in Table S2. Among the tested procedures, Protocol 4 [17] was the only one employing pure methanol as the extraction solvent and was therefore considered the most promising for co-extraction of the compounds with markedly different polarity. The protocol proposed by Zoidou et al. [15] was excluded because it relies on boiling water, which is unsuitable for the efficient solubilization of triterpenic acids. The method reported by Crawford et al. [16] was not selected due to the use of dimethyl sulfoxide (DMSO), a solvent with a high boiling point and poor biodegradability that additionally requires a subsequent solid-phase extraction (SPE) step, increasing analytical complexity and cost. Other reported protocols employing aqueous methanol (8:2, *v*/*v*) [13,29] were not considered, as they exclusively aimed at phenolic compound analysis and involved a substantial water content that could limit triterpenic acid extraction efficiency [12]. For comparative purposes, after extraction, the solvents were evaporated, and residues were reconstituted in methanol. Volumes were adjusted to yield solutions of uniform concentration (10,000 mg/L) across all protocols. Each protocol was applied in three independent extractions (*n* = 3), and each extract was analysed in triplicate. The resulting concentrations, expressed as mg/kg dry weight of raw olive flesh, are summarized in Table 2. Protocol 4 yielded significantly higher concentrations of tyrosol and of both triterpenic acids (1.28–1.53-fold higher than the next-best protocol; *p* < 0.05, as denoted by the superscript letters in Table 2). The magnitude of these differences was further confirmed by effect-size analysis, which yielded very large values for the triterpenic acids (Cohen’s d = 10.0 and 17.9 for maslinic and oleanolic acids, respectively, relative to Protocol 3), consistent with the low within-protocol variability. Conversely, it gave significantly lower concentrations for hydroxytyrosol compared with Protocol 2 and for oleuropein compared with Protocols 1–3 (*p* < 0.05); however, its markedly superior extraction of the triterpenic acids, together with its practical advantages, guided its selection. These results indicate that the combination of pure methanol with ultrasound-assisted extraction provided effective co-extraction of all target analytes. In addition, Protocol 4 offers practical advantages, including reduced solvent consumption, a single-step extraction requiring no repeated solvent additions or centrifugations, and the absence of an evaporation-reconstitution step (Table S2), all aligning with principles of green analytical chemistry. Consequently, Protocol 4 was selected for all subsequent method validation and application studies.

### 2.3. Method Validation

#### 2.3.1. Selectivity

Method selectivity was evaluated according to established validation principles for diode-array detection using complementary chromatographic, spectral, and quantitative criteria: retention time repeatability, peak purity, UV–Vis spectral matching with reference standards, and recovery experiments with authenticated standards, applied to the three targeted table olive matrices.

For phenolic compounds, selectivity was supported by detection at higher wavelengths (240 nm for oleuropein and 280 nm for hydroxytyrosol and tyrosol) and in-line fluorescence detection, with single, well-defined peaks were consistently observed.

**Figure 3:**
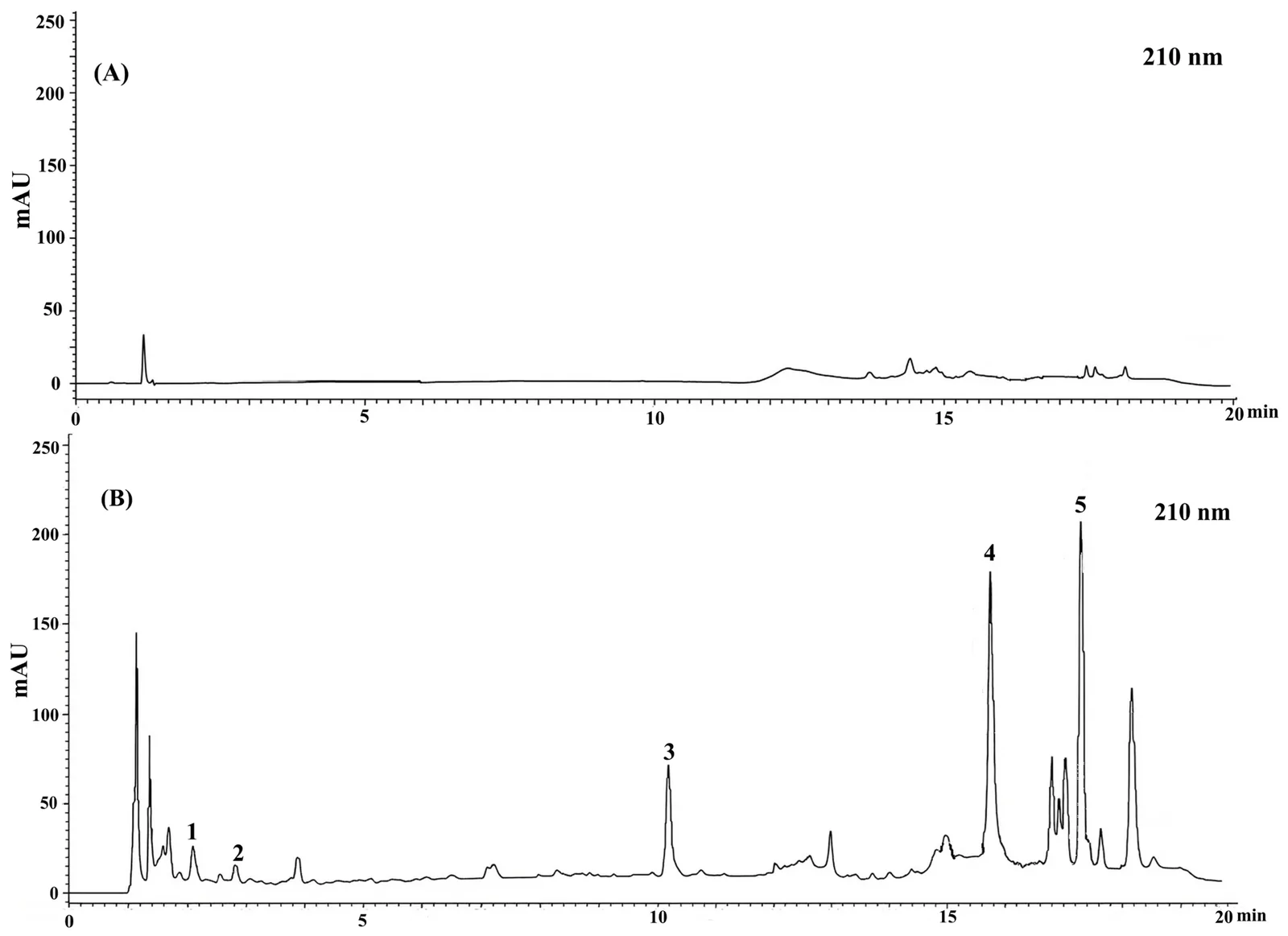
Chromatogram recorded at 210 nm for (**A**) solvent injection (blank) and (**B**) Throumba Thassou black olives. Peak identification: 1. hydroxytyrosol (2.063 min); 2. tyrosol (3.007 min); 3. oleuropein (10.138 min); 4. maslinic acid (15.768 min); 5. oleanolic acid (17.058 min).

For triterpenic acids (maslinic and oleanolic acids), detection was at 210 nm, a wavelength commonly used due to the absence of extended chromophores in these compounds, as in validated HPLC methods for botanicals [22,23], one of which similarly uses a phosphate-based mobile phase [23]. Although this region is inherently less selective, method performance was assessed through multiple complementary approaches. Retention times were highly reproducible across samples and analytical runs (relative standard deviation, %RSD 0.032–0.042%, *n* = 35). Diode-array evaluation showed peak purity indices of 0.973–1.000 for the triterpenic acids eluted in the more crowded region (Figure S3), with strong spectral similarity to authenticated standards (0.9767–0.9895, Figure S4), all exceeding the critical match factor of 0.95 established during method validation, in accordance with Commission Decision 2002/657/EC [30]. The absence of UV-active co-eluting interferences was further supported by applying second-derivative transformation to the chromatographic peaks of the triterpenic acids (Figure S5); no hidden shoulders or additional inflection points indicative of co-elution were observed, consistent with single-component peaks. A chromatogram of Throumba Thassou olives at 210 nm, together with a solvent blank (Figure 3), demonstrates baseline stability and adequate peak resolution (for the other two matrices, see Figure S2).

Peak purity was assessed against the analyte-specific single-point thresholds computed by the software (0.810–0.921); the purity indices obtained (0.973–1.000) exceeded these thresholds for all five analytes, indicating spectrally homogeneous peaks. Collectively, these complementary chromatographic, spectral, and quantitative assessments indicated that the proposed UHPLC-DAD method provides adequate selectivity for the targeted quantification of major phenolic compounds and triterpenic acids in table olives, consistent with the evaluation approaches described in the International Council for Harmonisation (ICH) Q2(R1) and Eurachem guidelines [31,32].

#### 2.3.2. Linearity, Limits of Detection and Quantification

Linearity was evaluated over analyte-specific concentration ranges (Table 3), and all calibration curves exhibited excellent linearity, with coefficients of determination (R^2^) > 0.999. In all cases, the regression sum of squares (SSREG) and mean square (MSREG) were substantially higher than the corresponding residual values with very low associated *p* values, confirming the adequacy of the linear model beyond the coefficient of determination alone [33].

The calculated limits of detection (LOD) and quantification (LOQ) are also reported in Table 3, expressed on a dry-weight basis. As expected, these are higher than values reported in validated LC–MS protocols for the same analytes [13,18,19,34]. Among HPLC-DAD methods, the present values were higher than those of Zoidou et al. [15] for hydroxytyrosol and oleuropein, but in good agreement with the validated method of Crawford et al. [16]. Differences among studies can be attributed to variations in extraction procedures, instrumental configuration, and LOD/LOQ calculation approaches. Overall, the method demonstrates satisfactory linearity and sensitivity for the intended quantitative analysis.

#### 2.3.3. Instrumental Accuracy and Precision

Accuracy and precision were evaluated using standard solutions of the target analytes across the working range, including concentrations at the LOQ (Table 4). Accuracy, expressed as relative error (RE), was consistently below 15% above the LOQ, indicating good agreement with nominal values. At the LOQ, an RE of 20% was observed only for tyrosol, which is within the limits of SANTE/11312/2021 [35] (±20% at LOQ) for pesticide residues, although not within the limits specified by AOAC International [36]. This elevated error at the lowest calibration level reflects the reduced precision inherent to concentrations approaching the LOQ; accordingly, quantification is considered reliable only at or above the LOQ of each analyte (Table 3), with lower values reported as <LOQ. In the samples examined, all analyte concentrations were well above their respective LOQs (Table 5). Precision, expressed as relative standard deviation (RSD), was ≤8.4%. Overall, the method demonstrates reliable quantitative performance at concentrations at or above the LOQ, a range that includes the levels found in table olives. Accuracy and precision of the complete analytical procedure, including sample preparation, were subsequently assessed in the three olive matrices (Section 2.3.4).

#### 2.3.4. Method Performance in Representative Table Olive Matrices

For the recovery experiment, one sample per matrix was analysed unspiked, and two samples per matrix were spiked at a low (25 mg/kg for hydroxytyrosol, tyrosol, and oleuropein; 50 mg/kg for maslinic and oleanolic acids) and at a high concentration level (500 mg/kg for all compounds). The actual values and recovery results are presented in Table 5. Mean recoveries for all analytes ranged from 70.8% to 108.9%.

In two of the three matrices, lower recovery values were observed at the higher spiking level. Nevertheless, all values complied with the 70–120% range recommended by SANTE/11312/2021 [35] and AOAC [36]. Owing to the wide range of endogenous concentrations among analytes and matrices (76–4279 mg/kg), the low spiking level represented a small relative addition for the most abundant analytes and should be interpreted with caution, whereas the high level provided a more reliable assessment of accuracy. Method precision was good, with repeatability (% S_r_) values ranging from 0.1% to 7.9%, well within the accepted limits. The consistent recoveries obtained across the three matrices, despite their different processing, confirm that the selected extraction—previously established for phenolic compounds in Spanish-style table olives [17] and here extended to the triterpenic acids and to naturally fermented and dry-salted products—is applicable to compositionally diverse table olive samples.

Comparable recoveries have been reported. Kalogiouri et al. [29] obtained 81.4–94% for phenolic compounds in PDO Kalamata olives, while Crawford et al. [16] observed slightly higher recoveries for tyrosol compared to hydroxytyrosol in Manzanilla olives, in agreement with the present trends. In contrast, Johnson et al. [13] reported markedly lower recoveries (18–75% at 100 μg/L and 48–94% at 1000 μg/L) for phenolic compounds in Spanish- and Greek-style olives. For triterpenic acids, Giménez et al. [18] obtained 94.4 ± 7.5% (maslinic acid) and 91.4 ± 6.2% (oleanolic acid) in cv. Marfil olives, while Zoidou et al. [15] reported 82.4–90.8% for oleuropein and 70.3–78.5% for hydroxytyrosol by HPLC–DAD. Notably, Zoidou et al. [15] is the only cited study employing DAD, the others relying on MS-based methods.

Hydroxytyrosol was the predominant phenolic compound in all samples. Chalkidiki green and Kalamata black olives contained high concentrations (1211 and 939.3 mg/kg, respectively), whereas Throumba Thassou olives showed considerably lower levels (124.9 mg/kg) and were instead characterized by the presence of oleuropein. This predominance is consistent with the formation of hydroxytyrosol through hydrolysis of endogenous phenolic precursors during processing. The determined concentrations are comparable with previously reported values. Blekas et al. [9], using freeze-dried samples and HPLC–DAD, reported (on a dry-weight basis) 254–761 mg/kg in Kalamata olives, 287–513 mg/kg in Spanish-style Chalkidiki green olives, and 6378 mg/kg in naturally black Thassou olives. Zoidou et al. [15] reported (on a dry-weight basis) 414 mg/kg in Spanish-style green olives and 105 mg/kg in Throumba Thassou olives, in agreement with the present cultivar-related trends. García et al. [10] reported 1005 and 1133 mg/kg wet weight in Manzanilla and Hojiblanca olives preserved in acidified brine; given the moisture contents of these fruits (68% and 73%), these correspond to approximately 3140 and 4200 mg/kg dry weight, exceeding the present levels. Romero et al. [11] analysed the juice and oil phases of packed table olives separately and likewise identified hydroxytyrosol as the predominant phenolic compound in naturally black Kalamata and Thassos olives; as their results were expressed on a juice volume basis, direct comparison is not possible. Moreno-González et al. [19], using LC–MS analysis with matrix-matched calibration, reported 480–510 mg/kg in Greek-style naturally fermented Arbequina olives and 200–390 mg/kg in Empeltre olives (wet weight). Overall, despite differences in analytical approach and result expression basis, these values confirm the considerable variability of hydroxytyrosol accumulation with cultivar, fermentation process, and harvest year.

Tyrosol showed similar distribution but was consistently detected at lower concentrations than hydroxytyrosol, ranging from 76.4 mg/kg in Throumba Thassou olives to 424.3 mg/kg in Kalamata olives, with 305.9 mg/kg in Chalkidiki olives. These values lie within the published variability for table olives. Blekas et al. [9] found tyrosol generally below 170 mg/kg dry weight, whereas Moreno-González et al. [19] reported lower levels in fermented Arbequina and Empeltre olives (16–39 and 3–32 mg/kg wet weight, respectively). García et al. [10] measured 78.6 and 79.1 mg/kg wet weight in Manzanilla and Hojiblanca olives preserved in acidified brine, corresponding to approximately 246 and 293 mg/kg dry weight given the moisture contents of these fruits—comparable with the value determined here for Chalkidiki green olives. Differences among studies may reflect cultivar characteristics and the extent of secoiridoid hydrolysis during fermentation. Oleuropein was detected exclusively in Throumba Thassou olives (919.4 mg/kg), confirming the phenolic profile of naturally matured olives; in fermented brined olives it is usually absent or present at very low levels due to hydrolysis, diffusion, and degradation during processing. Zoidou et al. [15] reported approximately 550 mg/kg wet weight in Throumba Thassou olives (~728 mg/kg dry weight after moisture normalization), comparable with the present value, while Melliou et al. [14] found higher levels (~1459 mg/kg wet weight; ~1932 mg/kg dry weight), reflecting the effect of cultivar, maturation stage, and processing on oleuropein retention.

Both maslinic and oleanolic acids were detected at substantial concentrations in all samples. Maslinic acid ranged from 1973.5 mg/kg in Chalkidiki to 4278.7 mg/kg in Throumba Thassou olives, with 3634.8 mg/kg in Kalamata olives. Oleanolic acid ranged from 1296.4 to 1583.8 mg/kg, highest in Kalamata olives. These results confirm that table olives are an important dietary source of pentacyclic triterpenic acids.

Previous studies report considerable variability in triterpenic acid concentrations depending on cultivar, processing, and analytical method. Giménez et al. [18], by LC–MS, found approximately 4800 and 3820 mg/kg dry weight of maslinic and oleanolic acids in the triterpene-rich Marfil cultivar. Romero et al. [12], analysing dried fruits, measured 287 to 1318 mg/kg (maslinic acid) and 169 to 841 mg/kg (oleanolic acid) in commercial table olives, while Alexandraki et al. [7], using the same extraction, obtained (on a dry-weight basis) 1349 and 536 mg/kg for naturally fermented Conservolea olives and 1260 and 706 mg/kg for naturally fermented Kalamon olives. García et al. [10] reported (on a dry-weight basis) 1550 and 450 mg/kg in Manzanilla olives and 2200 and 820 mg/kg in Hojiblanca olives, quantified against external standards. Moreno-González et al. [19], by LC–APCI–MS with internal standards and matrix-matched calibration, measured maslinic acid at 2417–2508 mg/kg (Greek-style naturally fermented Arbequina) and 1468–1862 mg/kg (Empeltre) olives, and oleanolic acid at 758–898 mg/kg; these values, however, are expressed on a wet-weight basis and are therefore not directly comparable.

The lower triterpenic acid contents in Chalkidiki green olives, relative to the two naturally fermented products, agree with the reported effect of alkaline treatment. Romero et al. [12] showed that NaOH debittering reduces maslinic and oleanolic acids through solubilisation into the alkaline solutions, with natural black olives retaining higher levels, while Alexandraki et al. [7] described a 55% decrease in maslinic acid after alkaline processing of Conservolea olives, unlike in naturally fermented fruits. With a single sample per product type, however, this observation cannot be attributed causally.

The present concentrations were higher than those obtained by Romero et al. [12], Alexandraki et al. [7], and García et al. [10] but comparable with those recently reported by Georgalaki et al. [37] for naturally fermented Greek Tsounati olives (4764 and 1807 mg/kg maslinic and oleanolic acids, respectively, on a dry-weight basis), and below those of Giménez et al. [18] for the triterpene-rich Marfil cultivar. Such differences may reflect cultivar, maturation stage, processing, and quantification procedures; nevertheless, the relative distribution among the three matrices examined is in line with the influence of the processing method on triterpenic acid retention reported previously [7,12].

Overall, the concentrations determined in this study fall within the ranges previously reported for comparable cultivars and processing conditions. Direct comparison remains inherently limited, however, not least because the basis on which results are expressed is not always stated; the comparisons presented above should therefore be regarded as indicative of the expected concentration ranges rather than as strict equivalence. Although the present results were not cross-validated against an MS-based method—the phosphate-based mobile phase required for detection at 210 nm being incompatible with ESI-MS—accuracy was established through recovery experiments and agreement with independently reported literature values, and analyte identity through peak purity, spectral similarity, and second-derivative analysis. Nonetheless, the results demonstrate the suitability of the validated UHPLC-DAD method as an accessible, fit-for-purpose alternative to MS-based approaches for the simultaneous determination of the major phenolic compounds and triterpenic acids in diverse commercial table olive matrices.

### 2.4. Metric Tools

#### 2.4.1. Evaluation of the Green Character of the Analytical Method (ComplexGAPI)

**Figure 4:**
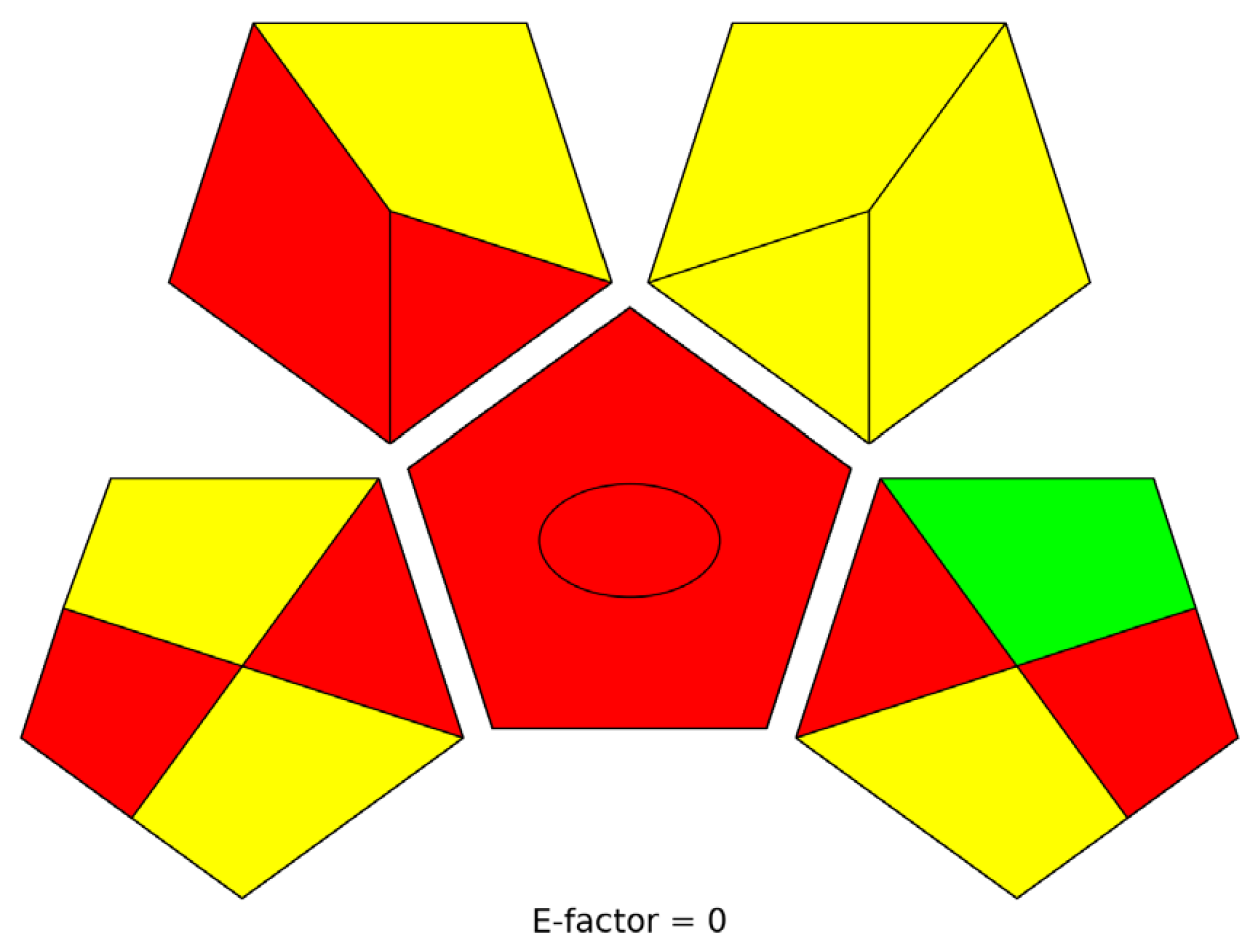
ComplexGAPI pictogram of the green assessment of the analytical method. Green, yellow and red fields denote environmentally favourable, moderately sustainable and less sustainable aspects of the procedure, respectively. The uncoloured pentagon and the E-factor of zero shown below the pictogram both reflect the absence of any pre-analytical synthesis step.

The environmental profile of the method was assessed using the ComplexGAPI [24], which provides a graphical representation of the ecological footprint of an analytical procedure; the individual criteria and the corresponding selections are listed in Table S3. The resulting pictogram (Figure 4) is dominated by yellow and red fields, the latter reflecting the off-line nature of the procedure, the extraction step required for the target analytes, the use of methanol, the volume of liquid waste generated, and the energy demand. The energy demand is dominated by freeze-drying (approximately 1.5 kWh per sample, against 0.2 kWh for the chromatographic analysis), a step that is nevertheless functionally necessary, as it minimises thermal degradation of the analytes, allows results to be expressed on a dry-weight basis, and limits water interference in the determination of the triterpenic acids (Section 3.1). As the method involves no pre-analytical synthesis, all fields of the pre-analysis pentagon were designated as not applicable, and the E-factor is zero.

Overall, the assessment indicates a procedure of moderate greenness, with scope for improvement through reduced organic solvent consumption and more efficient use of the freeze-drying capacity, which here was operated well below its nominal load.

#### 2.4.2. Evaluation of the Applicability of the Analytical Method (BAGI)

The practicality of the analytical method developed for the simultaneous determination of phenolic compounds and triterpenic acids in table olives (Chalkidiki green olives, Kalamata black olives, and Throumba Thassou black olives) was evaluated using the Blue Applicability Grade Index (BAGI), a recently introduced assessment tool in analytical chemistry.

The method enabled the quantification of five analytes belonging to two different chemical classes (three phenolic compounds and two triterpenic acids) using readily available reagents, simple instrumentation, and low-cost preparation. The analytical throughput was approximately 2.4 samples/h, while simultaneous preparation of approximately 12 samples was feasible. No preconcentration was required to achieve the necessary sensitivity, and sample preparation involved several conventional steps (extraction, centrifugation and filtration); freeze-drying, applied prior to extraction, was regarded as a sample-stabilisation and homogenisation step rather than analyte preconcentration. Only 2 g of sample was required, and the entire procedure was performed in a semi-automated mode (e.g., UHPLC autosampler).

**Figure 5:**
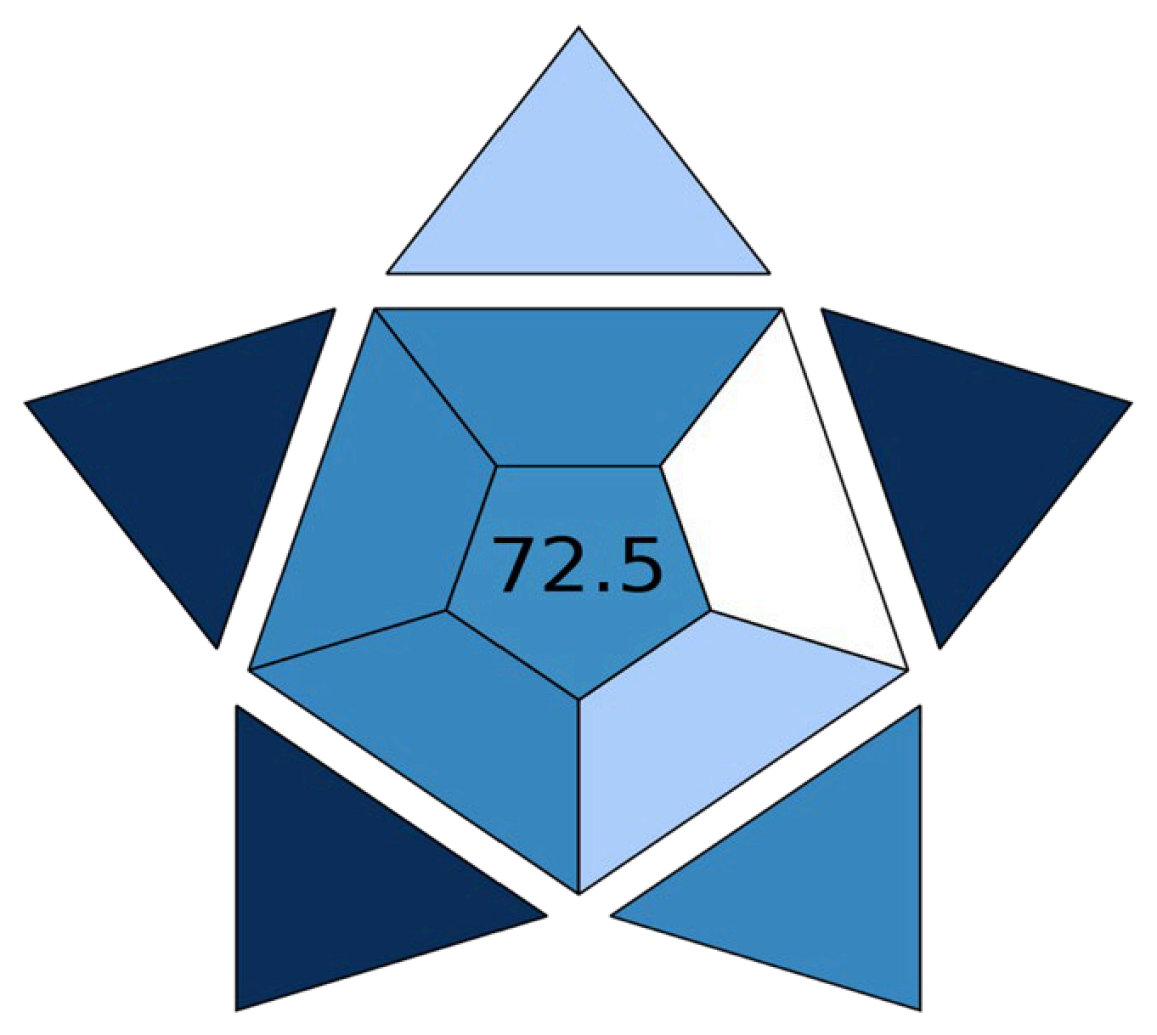
Assessment of the practicality of the analytical method based on the BAGI criteria. Darker blue shades indicate higher compliance with a given criterion; the overall score is shown at the centre of the pictogram.

Based on these criteria, a BAGI [24] score of 72.5 points (Figure 5) was assigned, indicating good practical applicability of the proposed method. The individual criterion scores are provided in Table S4, and even if freeze-drying were considered a preconcentration step, the score would remain 70, still denoting a readily applicable method.

## 3. Materials and Methods

### 3.1. Samples

Commercial samples representing three major Greek table olive cultivars were analysed: Chalkidiki green olives (Spanish-style; alkaline-treated green olives fermented in brine), Kalamata black olives (Greek-style; naturally mature black drupes fermented in brine without alkaline treatment), and Throumba Thassou black olives (naturally blackened, subjected to dry-salting). All commercial samples originated from the 2024 harvest, were purchased as pre-packaged, vacuum-sealed retail products from local markets in Thessaloniki, Greece (September 2024), and stored at 4 °C until analysis. Raw, unprocessed Chalkidiki olives from the 2024 harvest, available in the laboratory collection and containing all five target analytes at quantifiable levels, were used exclusively for comparative screening of the four extraction protocols (see Section 2.2). All samples were freeze-dried (−50 °C under vacuum, <0.1 mbar), pitted, and powdered before analysis. Freeze-drying was selected to: (i) minimize thermal degradation of bioactive compounds and improve storage stability, (ii) express results on a dry-weight basis, facilitating comparison with literature data and accounting for moisture variability among processing methods, and (iii) minimize potential water interference during triterpenic acid analysis [11].

### 3.2. Standards, Reagents, and Solvents

All solvents were HPLC grade. The standard compounds hydroxytyrosol (98%), oleuropein (80%), and maslinic acid (98%) were purchased from Biosynth-Carbosynth^®^ (Compton, Axis House, High Street, UK). Tyrosol (98%) and oleanolic acid (97%) were purchased from Alfa Aesar^®^ (Haverhill, MA, USA) and Sigma Aldrich^®^ (St. Louis, MO, USA), respectively. Methanol (99.8%), acetonitrile (99.9%), and water were purchased from CHEM-LAB^®^ (Zedelgem, Belgium), whereas phosphoric acid (85%) was purchased from Sigma Aldrich^®^ (St. Louis, MO, USA). Stock solutions were prepared by accurately weighing each standard and dissolving it in methanol. The oleuropein concentration was corrected for the certified purity of the standard (80%); the remaining standards, with purities ≥ 97%, were used without correction. Stock solutions were stored at −20 °C in amber vials, and working standards were prepared by serial dilution in methanol immediately before each analytical sequence.

### 3.3. Extraction Protocol

The extraction protocol selected after comparative screening (see Section 2.2) was adapted from Cabrera-Bañegil et al. [17] with minor modifications: olive sample powder (2.0 g, including both flesh and skin) was extracted with 10 mL of HPLC-grade methanol in a 100 mL conical flask. The mixture was sonicated in an ultrasonic bath (Elma S 30H, Elma Schmidbauer GmbH, Singen, Germany; 37 kHz, 80 W) for 30 min at ambient temperature without external temperature control. The water level was adjusted to a constant height and the bath water renewed before each extraction cycle, so that every extraction started from the same initial temperature and cumulative heating between successive cycles was avoided. The extract was subsequently centrifuged (Thermo Fisher Scientific, Waltham, MA, USA) at 1677× *g* for 10 min at 4 °C to promote phase separation. The resulting supernatant was filtered through a 0.22 μm PTFE membrane (Target Analysis, Pefka, Greece) prior to chromatographic analysis.

### 3.4. UHPLC-DAD Analysis

Chromatographic analysis was performed using a Shimadzu Nexera X2 UHPLC system (Shimadzu Corporation, Kyoto, Japan) equipped with a DGU-20ASR degasser, LC-30AD pump, SIL-30AC autosampler (50 μL loop), CBM-20A communication unit, CTO-20AC column oven, and SPD-M20A diode-array detector. Data acquisition and analysis were performed using LabSolution software v5.86 (Shimadzu Corporation, Kyoto, Japan).

Separation was achieved on an Accucore C18 column (100 × 4.6 mm, 2.6 μm; Thermo Fisher Scientific, Waltham, MA, USA) maintained at 35 °C using gradient elution. The mobile phase consisted of (A) 0.2% (*v*/*v*) aqueous phosphoric acid (resulting pH of ~2) and (B) acetonitrile, at a flow rate of 0.8 mL/min. The gradient was programmed as follows: 0–10 min, 10–25% B; 10–15 min, 25–95% B; 15–17 min, 95% B; 17–20 min, 10% B; re-equilibration 20–25 min. The sample temperature was maintained at 6 °C, and the injection volume was 1 μL. Detection wavelengths were set at 210 nm for triterpenic acids, 240 nm for oleuropein, and 280 nm for hydroxytyrosol and tyrosol, with spectra acquired over the range 190–400 nm. Peak integration was performed using the instrument software and verified manually for every peak, with baselines adjusted where necessary. For complementary purposes (see Section 2.3.1), a fluorescence detector (RF-20A XS) (Shimadzu Corporation, Kyoto, Japan) connected in series with the DAD was used and operated at λexc/λem: 280/320 nm.

### 3.5. Validation Procedures

Method validation was performed by assessing selectivity, linearity, limits of detection, and quantification (LOD/LOQ), precision, accuracy, and applicability. In the absence of harmonised validation criteria specific to endogenous bioactive constituents of foods, the performance criteria applied here were adopted from established guidelines for pesticide and veterinary drug residues [30,35] and from ICH Q2(R1) [31], Eurachem [32], and AOAC International [36] requirements. The residue-analysis guidelines [30,35] were developed for the trace-level determination of toxicologically relevant xenobiotics subject to statutory limits, and their acceptance criteria are therefore more demanding than would be required for endogenous constituents occurring at mg/kg levels. These criteria concern method performance characteristics (linearity, accuracy, precision, and selectivity) that are independent of the nature of the analyte. They are applied here as a conservative frame of reference and not to imply a regulatory context.

#### 3.5.1. Selectivity Assessment

Selectivity was evaluated using complementary chromatographic and spectral criteria, including retention time repeatability, peak purity assessment by photodiode-array (PDA) detection, UV–Vis spectral matching with reference standards, multi-wavelength monitoring, and evaluation of the calculated second derivative of chromatographic peaks. Retention time repeatability was assessed by calculating the relative standard deviation (%RSD) across replicate injections. Peak purity and spectral similarity indices were determined using PDA software (LabSolution v5.86; Shimadzu Corporation, Kyoto, Japan) to examine spectral homogeneity across each peak and agreement with authenticated standards. In addition, blank injections acquired under identical chromatographic conditions confirmed the absence of system- or solvent-related interferences.

#### 3.5.2. Linearity

Linearity was assessed using calibration standards prepared based on concentration ranges reported in the literature [6,8,11,14]. A mixed standard solution containing all analytes at approximately twice the average reported concentration (2N) was prepared, and serial dilutions in methanol were used to construct analyte-specific calibration curves. The lowest calibration level for each analyte was set at its calculated limit of quantification (LOQ), while the upper calibration level was selected to ensure linear detector response across the intended working range (Table 2). Each solution was analysed in triplicate on the same day, from the lowest to the highest concentration. Calibration curves were constructed by plotting the peak area of each individual injection (y) against analyte concentration (x). The regression equation, y = (a ± S_a_) + (b ± S_b_)x, and the coefficient of determination (R^2^) were calculated. Regression statistics, including analysis of variance of the regression, were examined to verify the adequacy of the linear model [33].

#### 3.5.3. Limits of Detection and Quantification

LOD and LOQ were estimated as $LOD=3.3\times \frac{s_{a}}{b},$ where s_a_ is the standard deviation of the y-intercept and b the slope of the curve. The calibration curves used for quantification were then constructed with their lowest level at the calculated LOQ of each analyte, so that the entire working range lies at or above the limit of quantification. The calculated LOQ values were verified experimentally by determining accuracy and precision at this lowest calibration level (Section 2.3.3); relative errors and relative standard deviations were within the ±20% criterion commonly applied at the limit of quantification.

#### 3.5.4. Instrumental Accuracy and Precision Using Standard Solutions

Precision and accuracy were evaluated by analysing standards at four concentration levels across the calibration range. Repeatability (intra-day, *n* = 6) and intermediate precision (inter-day, 5 consecutive days) were expressed as % relative standard deviation (% S_r_).

#### 3.5.5. Recovery Experiments in Representative Table Olive Matrices

Accuracy was assessed through recovery experiments conducted for each matrix. Freeze-dried, powdered samples were spiked on a dry weight basis at two levels (25 mg/kg for phenolic compounds, 50 mg/kg for triterpenic acids, and 500 mg/kg for all analytes) by adding the mixed standard solution directly to the powder, followed immediately by extraction, as described in Section 3.3. Spiked extracts were diluted as required to bring analyte concentrations within the respective calibration ranges. Spiking experiments were performed in triplicate. Recovery at each concentration level was calculated using the formula:

$$\%R=100\cdot \frac{\left(C_{spiked}-C_{blank}\right)}{C_{added}}$$

where *C*_spiked_ is the measured concentration in spiked samples, *C*_blank_ is the measured concentration in the unspiked (blank) samples, and *C*_added_ is the concentration of analyte added to the samples.

Robustness, the stability of standard solutions and extracts, autosampler stability, and carryover were not evaluated in the present validation. These parameters would be required before the method is implemented for routine quality control and remain to be addressed in further work.

### 3.6. Metric Tool Evaluation

The applicability of the developed UHPLC-DAD method for simultaneous determination of phenolic compounds and triterpenic acids in olives was evaluated using the Blue Applicability Grade Index (BAGI) [23]. Method greenness was further supported using the Complementary Green Analytical Procedure Index (ComplexGAPI), which considers both analytical methodology and sample preparation steps [24].

### 3.7. Statistical Analysis

Significant differences among mean values were assessed by one-way analysis of variance (ANOVA), followed by Tukey’s honestly significant difference (HSD) test (*p* < 0.05), using IBM SPSS Statistics 28.0.0.0 (IBM, Armonk, NY, USA). The independent experimental unit was the individual extraction (*n* = 3); injection replicates were averaged prior to analysis. Effect sizes (Cohen’s d) were additionally calculated for selected pairwise comparisons to complement significance testing. Linear regression analyses were performed in Microsoft Excel 2021 (Microsoft, Redmond, WA, USA).

## 4. Conclusions

A validated RP-UHPLC-DAD method was established for the simultaneous determination of phenolic compounds (hydroxytyrosol, tyrosol, and oleuropein) and triterpenic acids (maslinic and oleanolic acids) in three representative table olive substrates (Chalkidiki green olives, Kalamata black olives, and Throumba Thassou black olives). Method selectivity was demonstrated through peak purity assessment (0.973–1.000), UV spectral correlation, and chromatographic behaviour. The optimized methanolic extraction combined with rapid chromatographic separation provided satisfactory accuracy and precision, with recoveries of 70.8–108.9%, consistent with literature values, with hydroxytyrosol predominating in the Chalkidiki and Kalamata samples, whereas oleuropein was detected only in Throumba Thassou olives. ComplexGAPI indicated moderate greenness, with scope for further improvement, while the BAGI score of 72.5 denoted good practical applicability with minimal sample requirements. Overall, the method represents a reliable and cost-effective tool for routine quality control, process monitoring, and research on the nutritional and functional properties of table olives, offering an accessible alternative to MS-based approaches for targeted quantification of the major bioactive compounds. Future work could extend the validated method to a wider range of olive cultivars and processing styles, supporting the establishment of a reference database of phenolic and triterpenic acid levels in table olives.

## Figures and Tables

**Table 1:** System suitability parameters determined for a standard mixture of the five target analytes (USP convention).

Analyte	t_R_ (min)	R_s_ ^a^	Tailing Factor ^b^
Hydroxytyrosol	2.06	–	1.70
Tyrosol	3.01	5.4	1.92
Oleuropein	10.12	39.1	2.01
Maslinic acid	15.76	34.7	n.d.
Oleanolic acid	17.05	9.7	n.d.

^a^ Resolution relative to the preceding target analyte, calculated as R_s_ = 2(t_R2_ − t_R1_)/(W_b1_ + W_b2_). ^b^ USP tailing factor. n.d.: not determined; the narrow peak widths of the triterpenic acids precluded reliable measurement of the width at 5% peak height required by the USP formula. t_R_: retention time; R_s_: resolution.

**Table 2:** Levels of target analytes (mg/kg dry weight) in raw Chalkidiki green olives determined after application of the four extraction protocols.

Target Analytes					
(*n* = 3)	H-Tyr	Tyr	OE	MA	OA
	Protocol 1				
C_mean_ (mg/kg)	767.8 ^a^	481.5 ^a^	1073.1 ^b^	4830.3 ^a^	1427.8 ^a^
S (mg/kg)	18.9	14.3	65.5	236.6	49.6
% S_r_	2.5	3.0	6.1	4.9	3.5
	Protocol 2				
C_mean_ (mg/kg)	1208.8 ^c^	617.9 ^b^	1081.8 ^b^	4545.2 ^a^	1324.7 ^a^
S (mg/kg)	21.3	15.3	85.9	439.8	170.3
% S_r_	1.8	2.5	7.9	9.7	12.9
	Protocol 3				
C_mean_ (mg/kg)	754.9 ^a^	481.0 ^a^	1153.0 ^b^	4357.6 ^a^	1336.5 ^a^
S (mg/kg)	53.4	33.3	66.1	309.1	66.3
% S_r_	7.1	6.9	5.7	7.1	5.0
	Protocol 4				
C_mean_ (mg/kg)	1057.3 ^b^	895.1 ^c^	979.8 ^a^	6658.5 ^b^	2177.6 ^b^
S (mg/kg)	3.3	0.5	7.2	102.4	1.2
% S_r_	0.4	0.07	0.9	1.9	0.06

Values in the same column with different lowercase letters as superscripts are significantly different at *p* < 0.05. Each protocol was applied in three independent extractions (*n* = 3), each analysed in triplicate; injection replicates were averaged prior to statistical analysis. H-Tyr: hydroxytyrosol; Tyr: tyrosol; OE: oleuropein; MA: maslinic acid; OA: oleanolic acid. S: standard deviation. % S_r_: % relative standard deviation.

**Table 3:** Regression parameters, limits of detection (LOD), and limits of quantification (LOQ) of the target analytes in standard solutions.

Analyte	Linear Range (mg/L)	a (±S_a_)	b (±S_b_)	R^2^	SS_REG_/ SS_RES_	MS_REG_/ MS_RES_	*p*	LOD (mg/L)	LOD ^a^ (mg/kg)	LOQ (mg/L)	LOQ ^a^ (mg/kg)
H-Tyr	12.5–500	−2753.3 (±2019.7)	1653.9 (±9.1)	0.9992	1314.9	32,871.4	<1 × 10^−39^	4.03	20.1	12.21	61.1
Tyr	2.5–100	−595.5 (±314.2)	1165.9 (±7.1)	0.9991	1079.7	26,992.3	<1 × 10^−38^	0.89	4.4	2.70	13.5
OE	7.5–300	−670.5 (±291.1)	387.0 (±2.2)	0.9992	1247.4	31,184.1	<1 × 10^−39^	2.48	12.4	7.52	37.6
MA	15.0–1100	1443.5 (±1217.9)	794.5 (±2.8)	0.9996	2665.8	82,640.9	<1 × 10^−53^	5.06	25.3	15.33	76.6
OA	10.0–400	3994.2 (±815.6)	797.7 (±4.6)	0.9992	1200.2	30,006.2	<1 × 10^−39^	3.37	16.9	10.22	51.1

Calibration curves were constructed from all individual injections (*n* = 27 data points; 9 concentration levels × 3 injections) for H-Tyr, Tyr, OE, and OA, and *n* = 33 data points (10 levels × 3 injections) for MA. y = a + bx, where y is the peak area and x the concentration (mg/L). LOD = 3.3 × S_a_/b; LOQ = 10 × S_a_/b, where S_a_ is the standard error of the intercept and b the slope. ^a^ LOD and LOQ expressed on a dry-weight basis, calculated by applying the extraction factor (2.0 g of freeze-dried sample powder in 10 mL, i.e., × 5). H-Tyr: hydroxytyrosol; Tyr: tyrosol; OE: oleuropein; MA: maslinic acid; OA: oleanolic acid. S_a_: standard error of the intercept; S_b_: standard error of the slope; SS_REG_/SS_RES_: ratio of regression to residual sum of squares; MS_REG_/MS_RES_: ratio of regression to residual mean square.

**Table 4:** Accuracy and precision of the method using standard solutions of the target analytes.

Analyte	C (mg/L)	Intra-Day (*n* = 6)			Inter-Day (*n* = 6 × 5)		
		C_mean_ (mg/L)	% S_r_	% E_r_	C_mean_ (mg/L)	% S_r_	% E_r_
H-Tyr	12.5	14.12	0.2	13.0	14.50	3.3	16.0
	125	125.60	3.5	0.5	125.87	2.1	0.7
	250	237.80	0.2	−4.9	240.21	0.7	−3.9
	500	501.50	0.1	0.3	507.77	1.4	1.6
Tyr	2.5	2.96	1.6	18.4	2.96	4.4	18.4
	25	25.11	3.3	0.4	24.94	1.6	−0.2
	50	47.64	0.3	−4.7	47.96	0.5	−4.1
	100	100.80	0.2	0.8	101.80	1.3	1.8
OE	7.5	8.78	4.6	17.1	8.90	3.4	18.7
	75	76.39	3.4	1.9	75.84	1.8	1.1
	150	144.40	0.5	−3.7	144.80	0.6	−3.5
	300	303.30	0.3	1.1	307.30	1.6	2.4
MA	20	18.97	1.4	−5.2	20.07	8.3	0.5
	275	284.20	4.4	3.3	281.90	1.6	2.5
	550	532.60	0.4	−3.2	536.20	0.9	−2.5
	1100	1109.0	0.2	0.8	1121.0	1.3	1.9
OA	10	11.82	8.4	18.2	11.81	8.0	18.1
	100	100.90	5.2	0.9	101.20	6.0	1.2
	200	192.90	0.7	−3.6	193.00	3.0	−3.5
	400	407.60	1.8	1.9	408.10	1.9	2.0

C: nominal concentration; C_mean_: mean determined concentration. % S_r_: % relative standard deviation. % E_r_: % relative error = (C_mean_ − C)/C × 100. H-Tyr: hydroxytyrosol; Tyr: tyrosol; OE: oleuropein; MA: maslinic acid; OA: oleanolic acid.

**Table 5:** Precision and accuracy data in each substrate spiked at two concentrations.

Sample	Analyte	Initial Concentration (mg/kg)	% S_r_	Added Concentration (mg/kg)	Measured Concentration (mg/kg) (*n* = 3 × 3)	% S_r_	Recovery (%)
Chalkidiki green olives	H-Tyr	1211 ± 19.5	1.6	25	1230.2 ± 6.2	0.5	76.7
		1211		500	1573.5 ± 1.6	0.1	72.5
	Tyr	305.9 ± 6.9	2.2	25	327.4 ± 3.9	1.2	85.9
		305.9		500	714.9 ± 2.1	0.3	81.8
	OE	-					
	MA	1973.5 ± 96.3	4.9	50	2010.0 ± 22.1	1.1	72.9
		1973.5		500	2381.0 ± 4.8	0.2	81.5
	OA	1296.4 ± 46.6	3.5	50	1344.8 ± 9.4	0.7	96.8
		1296.4		500	1697.4 ± 1.7	0.1	80.2
Kalamata black olives	H-Tyr	939.3 ± 12.3	1.3	25	966.5 ± 6.8	0.7	108.9
		939.3		500	1375.3 ± 1.4	0.1	87.2
	Tyr	424.3 ± 3.0	0.7	25	442.0 ± 3.5	0.8	70.8
		424.3		500	804.3 ± 1.6	0.2	76.0
	OE	-					
	MA	3634.8 ± 180	4.9	50	3683.6 ± 11.1	0.3	97.6
		3634.8		500	3994.3 ± 8.0	0.2	71.9
	OA	1583.8 ± 65	4.1	50	1636.3 ± 14.7	0.9	104.9
		1583.8		500	1954.8 ± 3.9	0.2	74.2
Throumba Thassou black olives	H-Tyr	124.9 ± 4.8	3.8	25	149.1 ± 4.6	3.1	96.8
		124.9		500	494.4 ± 4.4	0.9	73.9
	Tyr	76.4 ± 0.8	1.1	25	97.0 ± 7.7	7.9	82.5
		76.4		500	464.9 ± 4.2	0.9	77.7
	OE	919.4 ± 35.2	3.8	25	940.8 ± 24.5	2.6	85.4
		919.4		500	1280.4 ± 11.5	0.9	72.2
	MA	4278.7 ± 11.4	0.3	50	4323.1 ± 8.6	0.2	88.8
		4278.7		500	4644.2 ± 27.9	0.6	73.1
	OA	1401.5 ± 6.9	0.5	50	1445.5 ± 30.4	2.1	87.9
		1401.5		500	1884.5 ± 9.4	0.5	96.6

H-Tyr: hydroxytyrosol; Tyr: tyrosol; OE: oleuropein; MA: maslinic acid; OA: oleanolic acid. % S_r_: % relative standard deviation.

## Data Availability

The data presented in this study are available in the article and in the Supplementary Materials; further data are available on request from the corresponding author.

## Supplementary Materials

The following supporting information can be downloaded at: https://www.mdpi.com/article/10.3390/molecules31173029/s1, Table S1: Physicochemical properties of target analytes in table olives [38,39,40,41]; Table S2: Comparison of four extraction protocols evaluated for simultaneous extraction of phenolic compounds and triterpenic acids from table olives; Table S3: ComplexGAPI assessment of the proposed RP-UHPLC-DAD method: criteria, selected options, and corresponding field assignments; Table S4: Blue Applicability Grade Index (BAGI) assessment of the proposed RP-UHPLC-DAD method: individual criteria and selected options; Figure S1: Chromatogram of a standard mixture containing the five target analytes recorded at 210 nm using the adapted gradient elution protocol of Xie et al. [21]; Figure S2: Chromatograms at 210 nm for (A) Chalkidiki green olives, (B) Kalamata black olives. Peak identification: 1. hydroxytyrosol (2.063 min); 2. tyrosol (3.007 min); 3. oleuropein (10.138 min); 4. maslinic acid (15.768 min); and 5. oleanolic acid (17.058 min); Figure S3: Peak purity indices of chromatographic peaks corresponding to maslinic and oleanolic acids in table olive sample extracts obtained by diode-array detection; Figure S4: UV spectral similarity indices comparing reference standards with corresponding sample peaks for triterpenic acids; Figure S5: Calculated 2nd derivative of the chromatogram (region of triterpenic acids) recorded at 210 nm for (a) Chalkidiki green olives, (b) Kalamata black olives, and (c) Throumba Thassou black olives. Negative peak identification: 4. maslinic acid (15.768 min); 5. oleanolic acid (17.058 min).
