# Supplementary material for: Simultaneous Determination of the Main Phenolic Compounds and Triterpenic Acids in Table Olives Using RP-UHPLC-DAD Chromatography
Source: Molecules. 2026 Aug 28;31(17):3029. doi: 10.3390/molecules31173029 (PMC13567158; doi:10.3390/molecules31173029)
Supplement: Supplementary file 1 [file molecules-31-03029-s001.zip › molecules-4482877-supplementary.pdf]

SUPPLEMENTARY MATERIAL

Simultaneous determination of the main phenolic compounds and triterpenic acids in table olives using RP-UHPLC-DAD chromatography.

Aggeliki Kalogeropoulou, Vasileios Zavrakoglou, Fani Th. Mantzouridou, Nikolaos Nenadis*

Laboratory of Food Chemistry & Technology, School of Chemistry, Aristotle University of Thessaloniki, 541 24 Thessaloniki, Greece

*Corresponding author:

Nikolaos Nenadis, E-mail address: niknen@chem.auth.gr

Table S1. Physicochemical properties of target analytes in table olives.

Compound	Molecular structure	MW (g/mol)	Water Solubility (mg/L)	pKa[a] (25°C)	Log *P*
Bioactive phenols					
Hydroxytyrosol (**H-Tyr**)	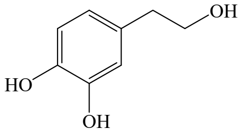	154.16	17.4[a]	9.46[b]	0.13[b]
Tyrosol (**Tyr**)	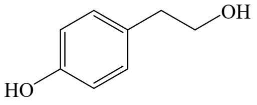	138.16	25.3[a]	10.20[c]	0.85[c]
Oleuropein (**OE**)	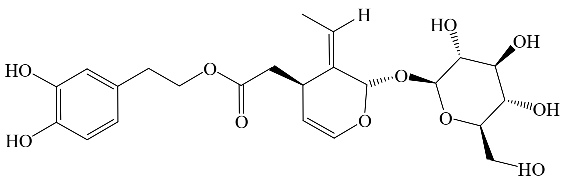	540.50	0.73[a]	9.28[b]	0.63[b]
Triterpenic acids					
Maslinic (**MA**)	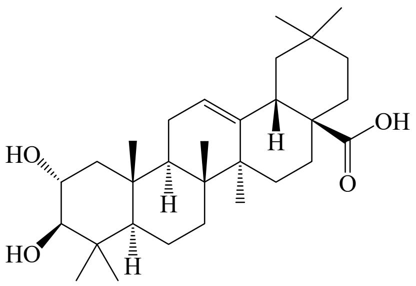	472.70	0.004[a]	4.74[b]	6.06[b]

Oleanolic (**OA**)	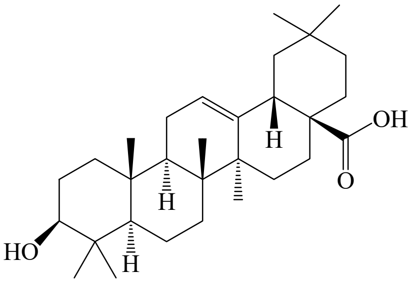	456.70	negligible[d]	2.52[d]	8.58[d]

a Literature values calculated using ALOGPS (Tetko & Tanchuk, [38]);

b Data retrieved from PhytoHub [39];

c Data retrieved from DrugBank (Knox et al., [40]);

d Data retrieved from ChemicalBook [41].

Table S2. Comparison of four extraction protocols evaluated for simultaneous extraction of phenolic compounds and triterpenic acids from table olives.

**Parameter**	**Protocol 1 Romero et al. [12]**	**Protocol 2 Papoti & Tsimidou, [28]**	**Protocol 3 Xie et al. [21]**	**Protocol 4 Cabrera-Bañegil et al. [17] (Selected)**
**Primary application**	Triterpenic acids	Phenolic compounds	Phenolic compounds	Phenolic compounds
**Sample amount**	1.0 g	0.5 g	3.0 g	2.0 g
**Extraction solvent**	Methanol:Ethanol (1:1, v/v)	Methanol	Ethanol:Water (9:1, v/v)	Methanol
**Solvent volume**	4 mL	20 mL	90 mL	10 mL
**Sample:solvent ratio**	1:4 (w/v)	1:40 (w/v)	1:30 (w/v)	1:5 (w/v)
**Extraction method**	6 successive extractions (1 min vortex each)	Ultrasound (5 min) + shaker (48 h)	Ultrasound (30 min)	Ultrasound (30 min)
**Total extraction time**	6 min (+ six centrifugation and separation steps)	48 h	30 min	30 min
**Centrifugation**	Yes (9500 rpm 5 min, 20 °C (per cycle)	Not required (filtered only)	Not specified	1677 × g, 10 min, 4°C
**Post-treatment**	Evaporation to dryness+ redissolution in solvent	Evaporation to dryness + reconstitution in methanol	Evaporation + redissolution in methanol	Direct filtration (0.22 µm PTFE)

Table S3. ComplexGAPI assessment of the proposed RP-UHPLC-DAD method: criteria, selected options and corresponding field assignments

	Criterion	Selected option	Field
1	Sample collection	Off-line	Red
2	Sample preservation	Chemical or physical (refrigeration; freeze-drying)	Yellow
3	Transport	Required	Red
4	Storage	Under normal conditions	Yellow
5	Type of method: direct or indirect	Extraction required	Red
6	Scale of extraction	Macro-extraction (2 g sample / 10 mL solvent)	Red
7	Solvents/reagents used	Non-green (methanol)	Red
8	Additional treatments	Simple (centrifugation, filtration)	Yellow
9	Amount of reagents/solvents	10–100 mL (≈17 mL organic solvent per sample)	Yellow
10	Health hazard	NFPA 2 or 3 (methanol, acetonitrile)	Yellow
11	Safety hazard	NFPA flammability/instability 2 or 3	Yellow
12	Energy consumption	> 1.5 kWh per sample	Red
13	Occupational hazard	Hermetic sealing of the process (fume hood)	Green
14	Waste	> 10 mL per sample (≈30 mL)	Red
15	Waste treatment	Degradation/passivation (licensed contractor)	Yellow
16	Type of analysis	Qualitative and quantitative	Symbol

Pre-analysis process: the method involves no pre-analytical synthesis or purification step; all criteria of the pre-analysis pentagon (yield, temperature–time conditions, reagents and solvents, health and safety hazards, technical set-up, energy, occupational hazard, work-up and purification, product purity) were therefore designated as not applicable, and the E-factor is zero.

Energy consumption was estimated from the rated power of the freeze-dryer (Christ Alpha 1-2 LD; 230 V, 3.5 A, 0.8 kVA) operated for 24 h together with its vacuum pump, for up to nine samples per cycle (≈1.5 kWh per sample), plus the chromatographic analysis (≈0.2 kWh per sample). Solvent consumption per sample comprises 10 mL of methanol for extraction and ≈7 mL of acetonitrile in the mobile phase (single injection, 0.8 mL min^{-1}, 25 min).

Criterion 16 is not colour-coded: in the ComplexGAPI pictogram a circle within the central field denotes a method providing both qualitative and quantitative information; the symbol is absent for purely qualitative methods.

The assessment describes the method as applied in routine analysis (single injection per sample); the replicate injections used in this work served validation purposes.

Table S4. Blue Applicability Grade Index (BAGI) assessment of the proposed RP-UHPLC-DAD method: individual criteria and selected options

#	**Criterion**	**Selected option**
1	Type of analysis	Quantitative
2	Multi-/single-element analysis	Multi-element analysis (2–15 analytes of different chemical classes)
3	Analytical technique	Simple instrumentation available in most laboratories (HPLC-DAD)
4	Simultaneous sample preparation	2–12 samples
5	Sample preparation	Multistep preparation
6	Analytical throughput	2–4 samples per hour
7	Reagents and materials	Common, commercially available reagents (e.g. methanol)
8	Preconcentration	No preconcentration required
9	Degree of automation	Semi-automated (e.g. autosampler)
10	Amount of sample	< 10 mL (or g) for food/environmental samples
	**BAGI score**	**72.5**

The assessment was performed using the online BAGI tool (bagi-index.anvil.app). The corresponding asteroid pictogram is shown in Figure 5. Freeze-drying was considered a sample-stabilisation and homogenisation step rather than analyte preconcentration; if treated as a preconcentration stage, the resulting score would be 70.

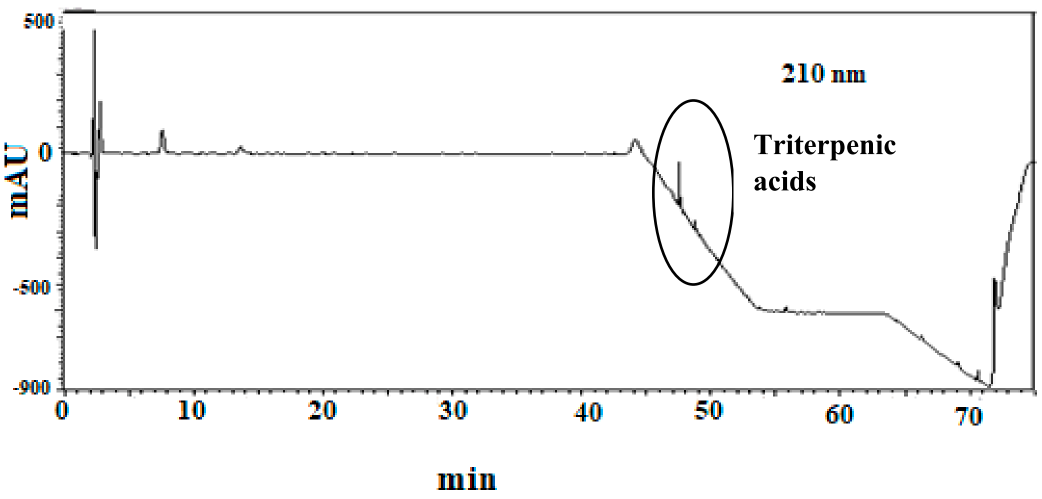

Figure S1. Chromatogram of a standard mixture containing the five target analytes recorded at 210 nm using the adapted gradient elution protocol of Xie et al. [21].

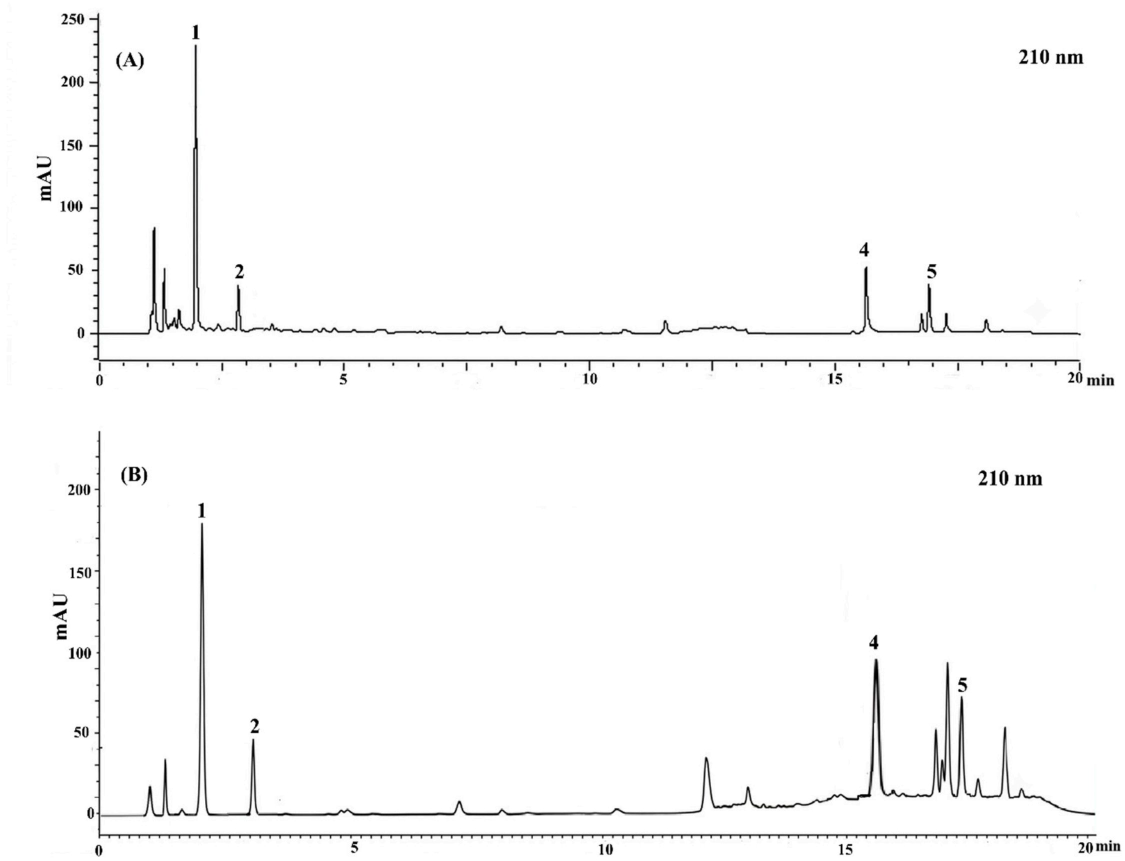

Figure S2. Chromatograms at 210 nm for (A) Chalkidiki green olives and (B) Kalamata black olives. Peak identification: 1. Hydroxytyrosol (2.063 min); 2. Tyrosol (3.007 min); 3. Oleuropein (10.138 min); 4. Maslinic acid (15.768 min); 5. Oleanolic acid (17.058 min).

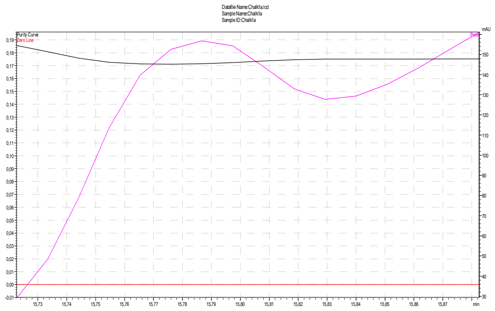

(MA/sample Chalkidiki) peak purity 1.000

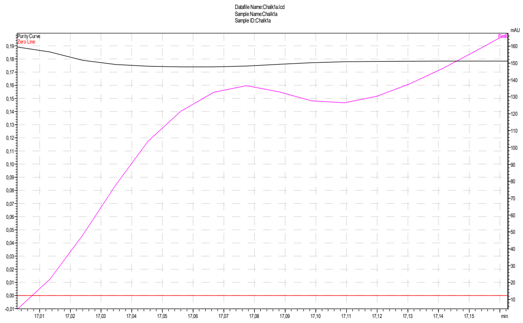

(OA/sample Chalkidiki) peak purity 1.000

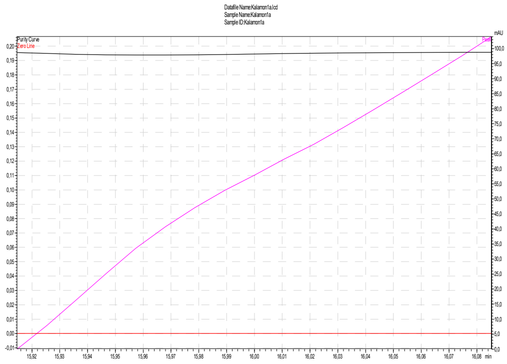

(MA/sample Kalamata) peak purity 1.000

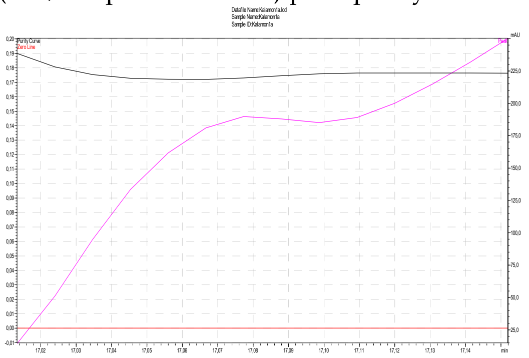

(OA/sample Kalamata) peak purity 0.999

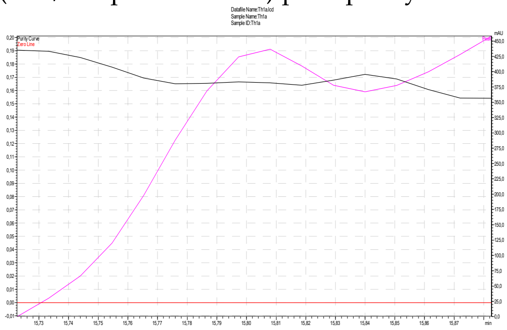

(MA/sample Throumba Thassos) peak purity 0.973

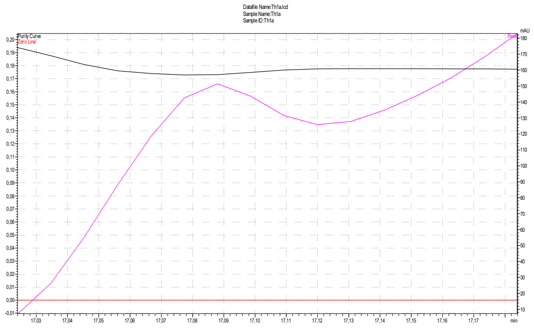

(OA/sample Throumba Thassos) peak purity 0.999

Figure S3. Peak purity indices of chromatographic peaks corresponding to maslinic and oleanolic acids in table olive sample extracts obtained by diode-array detection.

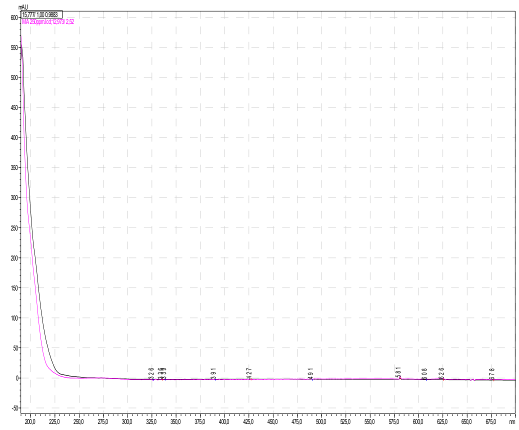

(MA/sample Chalkidiki) similarity index 0.9883

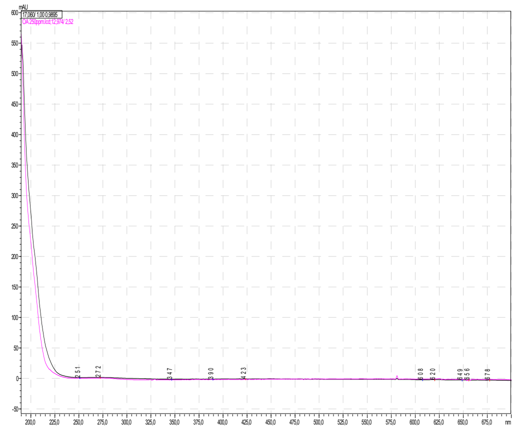

(OA/sample Chalkidiki) similarity index 0.9895

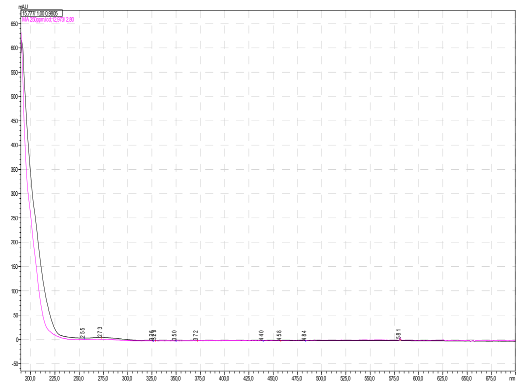

(MA/sample Kalamata) similarity index 0.9805

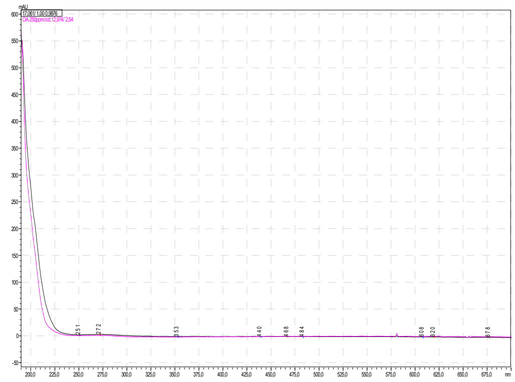

(OA/sample Kalamata) similarity index 0.9876

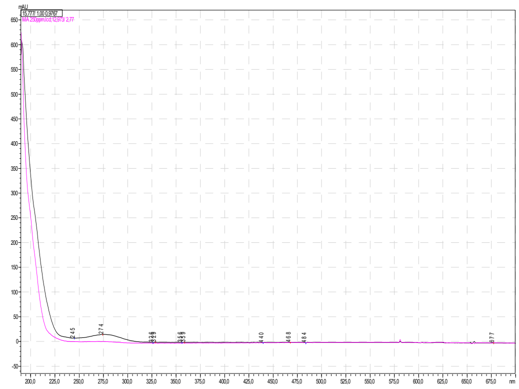

(MA/sample Throumba Thassos) similarity index 0.9767

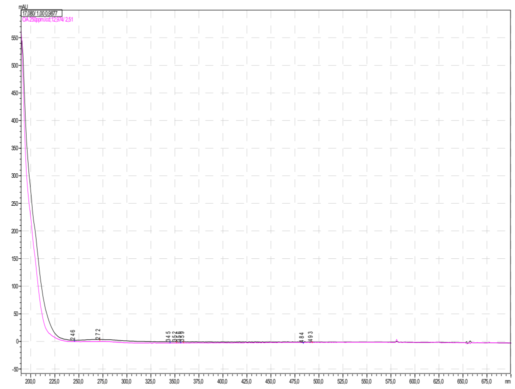

(OA/sample Throumba Thassos) similarity index 0.9877

Figure S4. UV spectral similarity indices comparing reference standards with corresponding sample peaks for triterpenic acids.

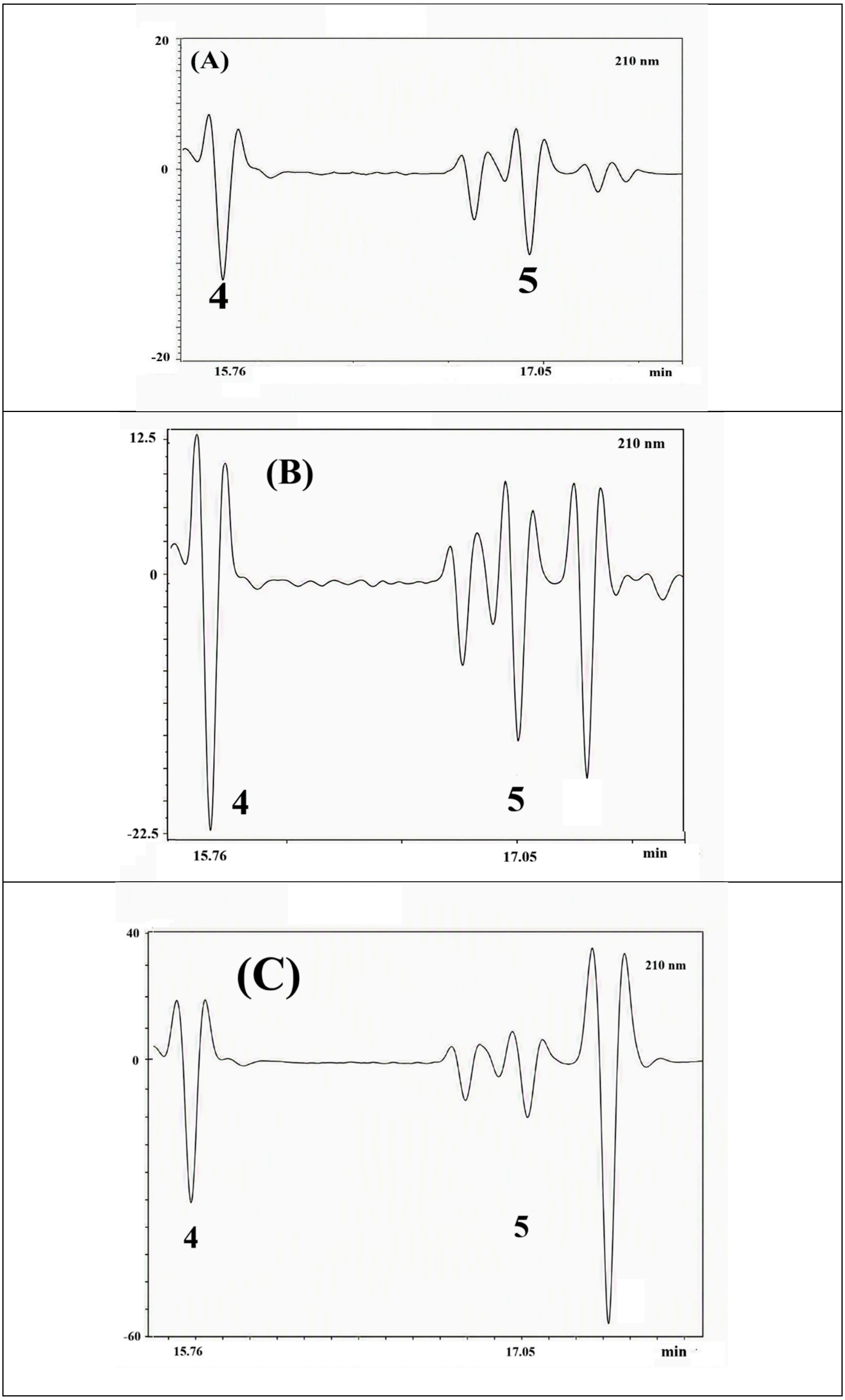

Figure S5. Calculated 2nd derivative of the chromatogram (region of triterpenic acids) recorded at 210 nm for (a) Chalkidiki green olives, (b) Kalamata black olives, and (c) Throumba Thassou black olives. Negative peak identification: 4. Maslinic acid (15.768 min); 5. Oleanolic acid (17.058 min).
